# Supplementary material for: Differential associations between neocortical tau pathology and blood flow with cognitive deficits in early-onset vs late-onset Alzheimer’s disease
Source: Eur J Nucl Med Mol Imaging. 2022 Jan 8;49(6):1951–63. doi: 10.1007/s00259-021-05669-6 (PMC9016024; doi:10.1007/s00259-021-05669-6)
Supplement: Supplementary file 1 — Supplementary file1 (DOCX 4350 KB) [file 259_2021_5669_MOESM1_ESM.docx]

**SUPPLEMENT**

Differential associations between neocortical tau pathology and blood flow with cognitive deficits in early-onset vs late-onset Alzheimer's disease

Denise Visser^1,†^, Sander CJ Verfaillie^1,†^, Emma E Wolters^1,2^, Emma M Coomans^1^, Tessa Timmers^1,2^, Hayel Tuncel^1^, Ronald Boellaard^1^, Sandeep SV Golla^1^, Albert D Windhorst^1^, Philip Scheltens^2^, Wiesje M van der Flier^2,4^, Bart NM van Berckel^1^ and Rik Ossenkoppele^2,3^

*^1^ Department of Radiology & Nuclear Medicine, Amsterdam Neuroscience, Vrije Universiteit Amsterdam, Amsterdam UMC, Amsterdam, The Netherlands*

*^2^ Alzheimer Center Amsterdam,^,^ Department of Neurology, Amsterdam Neuroscience, Vrije Universiteit Amsterdam, Amsterdam UMC, Amsterdam, The Netherlands
^3^ Clinical Memory Research Unit, Lund University, Lund, Sweden*

*^4^ Department of Epidemiology and Biostatistics, Vrije Universiteit Amsterdam, Amsterdam UMC, Amsterdam, The Netherlands*

*† both authors contributed equally*

Corresponding author:

Denise Visser, Department of Radiology & Nuclear Medicine, Amsterdam University Medical Centers, location VUmc, Amsterdam, The Netherlands, P.O. Box 7057, 1007 MB Amsterdam, The Netherlands; phone number: +31 20 4449298; fax number: +31 20 4448529. E-mail: [d.visser2@amsterdamumc.nl](mailto:d.visser2@amsterdamumc.nl); ORCID ID: 0000-0002-3642-146X

|  | EOAD | LOAD | p-values |
| --- | --- | --- | --- |
| Sample size (n) | 30 | 42 |  |
| Age (years) | 59 (5) | 71 (5) | <0.001 |
| Females n (%) | 14 (47) | 19 (45) | ̴1 |
| Education (Verhage scale*) | 6 [3-7] | 5 [3-7] | 0.637 |
| MMSE | 23 (3) | 23 (4) | 0.708 |
| APOE4 ε4 carriership, n/n_total_ | 21/30 | 33/38 | 0.757 |
| [^18^F]flortaucipir BP_ND_  Medial temporal  Lateral temporal  Parietal  Occipital  Frontal | 0.24 (0.14)  0.55 (0.31)  0.84 (0.51)  0.64 (0.54)  0.38 (0.30) | 0.25 (0.18)  0.42 (0.30)  0.33 (0.29)  0.29 (0.23)  0.16 (0.23) | 0.842  0.081  <0.001  <0.001  <0.001 |
| [^18^F]flortaucipir R_1_  Medial temporal Lateral temporal  Parietal  Occipital  Frontal | 0.69 (0.04)  0.86 (0.06)  0.86 (0.09)  0.97 (0.09)  0.89 (0.06) | 0.66 (0.05)  0.84 (0.07)  0.87 (0.08)  0.97 (0.08)  0.87 (0.06) | 0.004  0.155  0.794  0.891  0.209 |

**sTable-1. Demographics, [^18^F]flortaucipir BP_ND_ and R_1_ of n=72 (cognition) sample.** Depicted are mean (SD), unless specified otherwise, for early-onset AD (EOAD) and late-onset AD (LOAD) groups. Median [range] is depicted for education. APOE ε4 status was unknown for four LOAD subjects. Independent sample T-test or χ2 test was used for demographic variables. Differences in [^18^F]flortaucipir BP_ND_ or R_1_ were assessed using ANOVA, adjusted for sex. *The Dutch Verhage scale for education includes 7 ascending categories, ranging from one (representing less than six years of primary education) to 7 (representing a university degree).


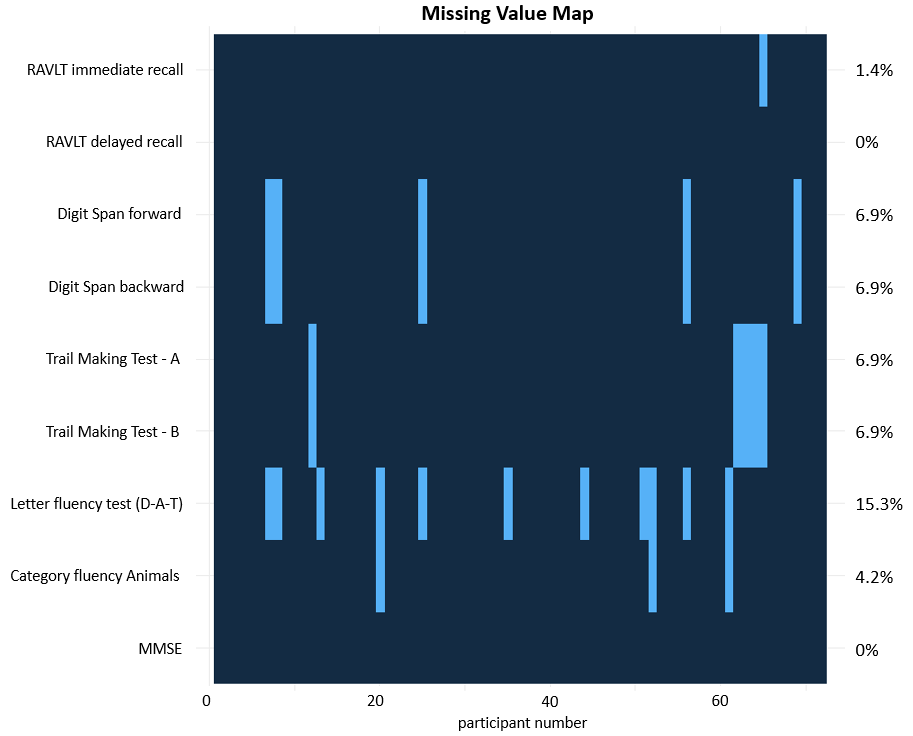


**sFigure-1. Map of missing values prior to imputation in the cognition-subsample.**


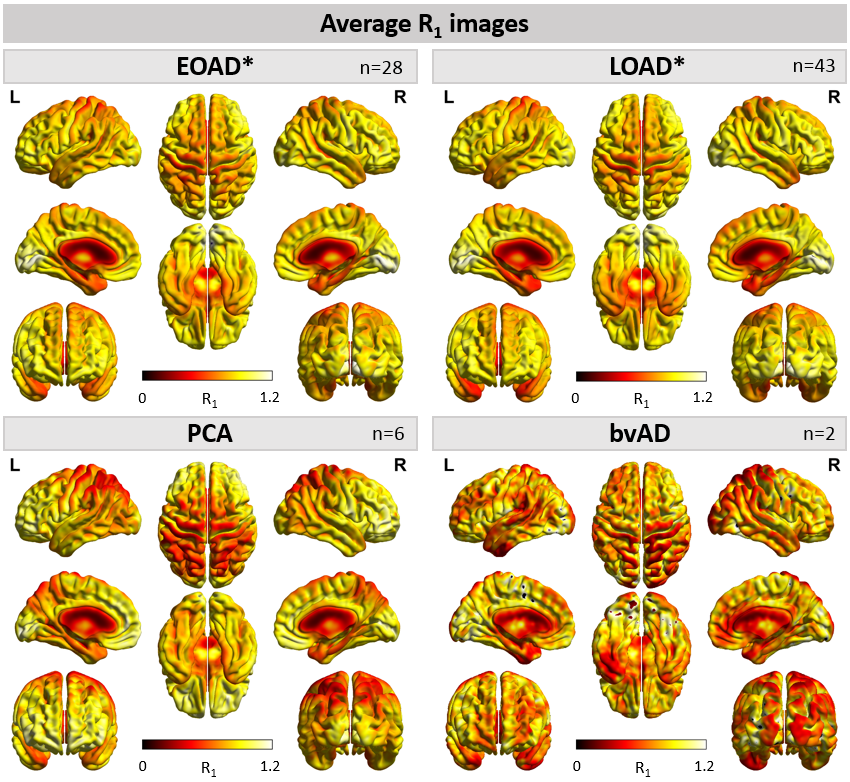


**sFigure-2. Average [^18^F]flortaucipir R_1_ images for early- and late-onset AD, PCA and bvAD.** Average images of all early-onset Alzheimer’s disease (EOAD), late-onset AD (LOAD), posterior cortical atrophy (PCA) patients and behavioral variant AD (bvAD) patients on a scale ranging from R_1_ 0 to 1.2. Low R_1_ values represent low relative cerebral blood flow.

* Excluding atypical variants, posterior cortical atrophy (PCA) patients and behavioral variant AD (bvAD) patients.


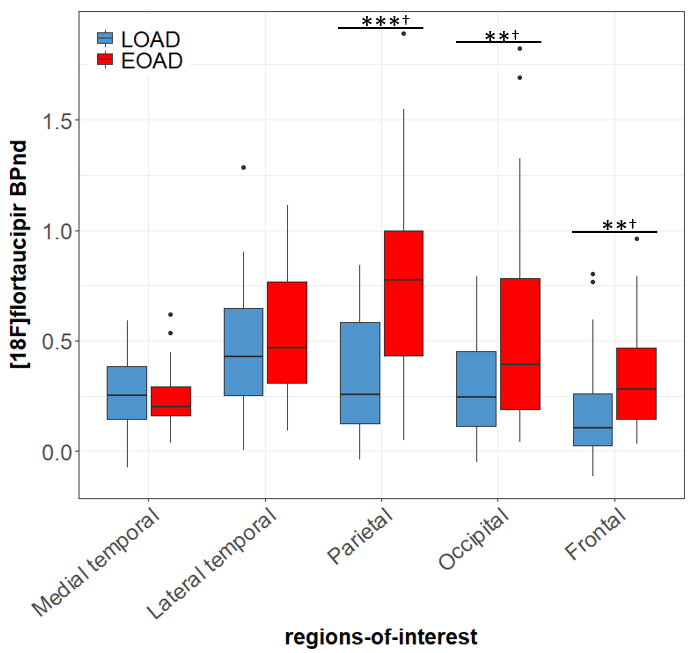


**sFigure-3. [^18^F]flortaucipir BP_ND_ for all subjects, excluding atypical (6 PCA and 2 bvAD) AD cases.** Differences between early-onset (EOAD) and late-onset AD (LOAD) were assessed using ANOVA, adjusted for sex. *p<0.05, ^**^p<0.01, ***p<0.001, †p_FDR_<0.05.


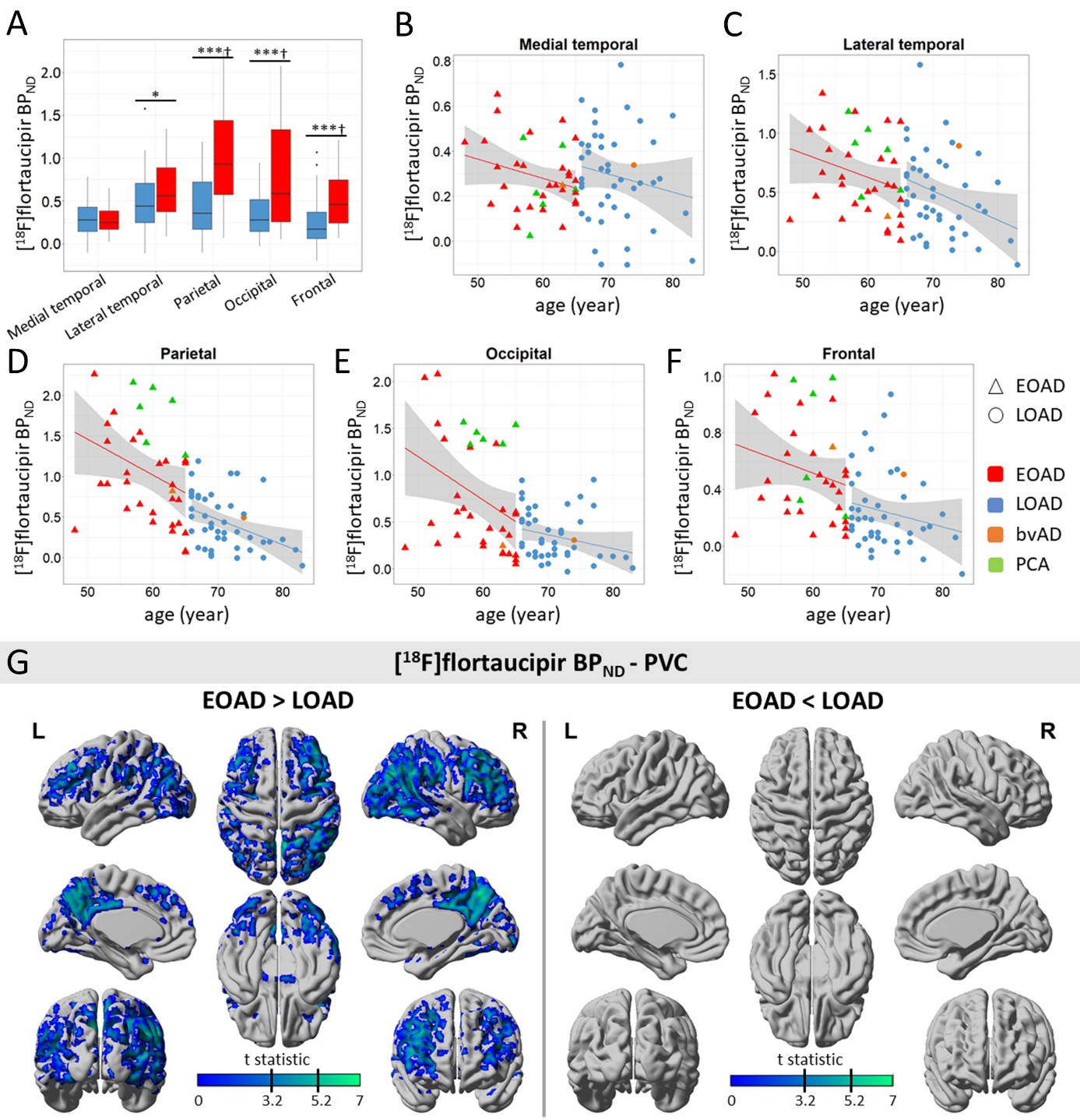


**sFigure-4. Partial volume-corrected (PVC) [^18^F]flortaucipir BP_ND_ for early- and late-onset AD**. A) Boxplot of partial volume-corrected [^18^F]flortaucipir BP_ND_ values for each region-of-interest (ROI). Differences were assessed using ANOVA, adjusted for sex. B-F) Scatterplots for partial volume-corrected [^18^F]flortaucipir BP_ND_ by age for each ROI. G) Results from voxel-wise contrast for partial volume-corrected [^18^F]flortaucipir BP_ND_ between early-onset AD (EOAD) and late-onset AD (LOAD). Indicated by the black lines on the color scale are thresholds for p<0.001, uncorrected (t = 3.20) and for p<0.05, FWE-corrected (t = 5.24). *p<0.05, **p<0.01, ***p<0.001, †p_FDR_<0.05.


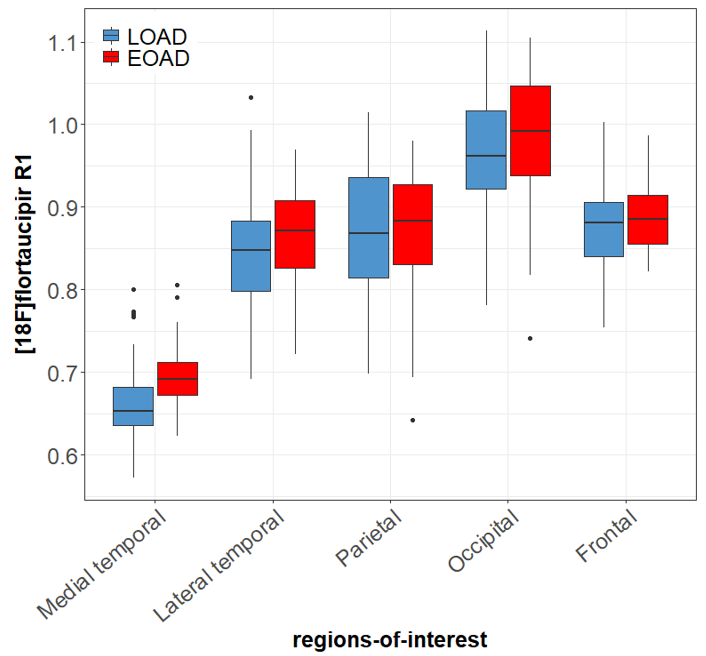


**sFigure-5. [^18^F]flortaucipir R_1_ for all subjects, excluding atypical (6 PCA and 2 bvAD) AD cases.** Differences between early-onset (EOAD) and late-onset AD (LOAD) were assessed using ANOVA, adjusted for sex.


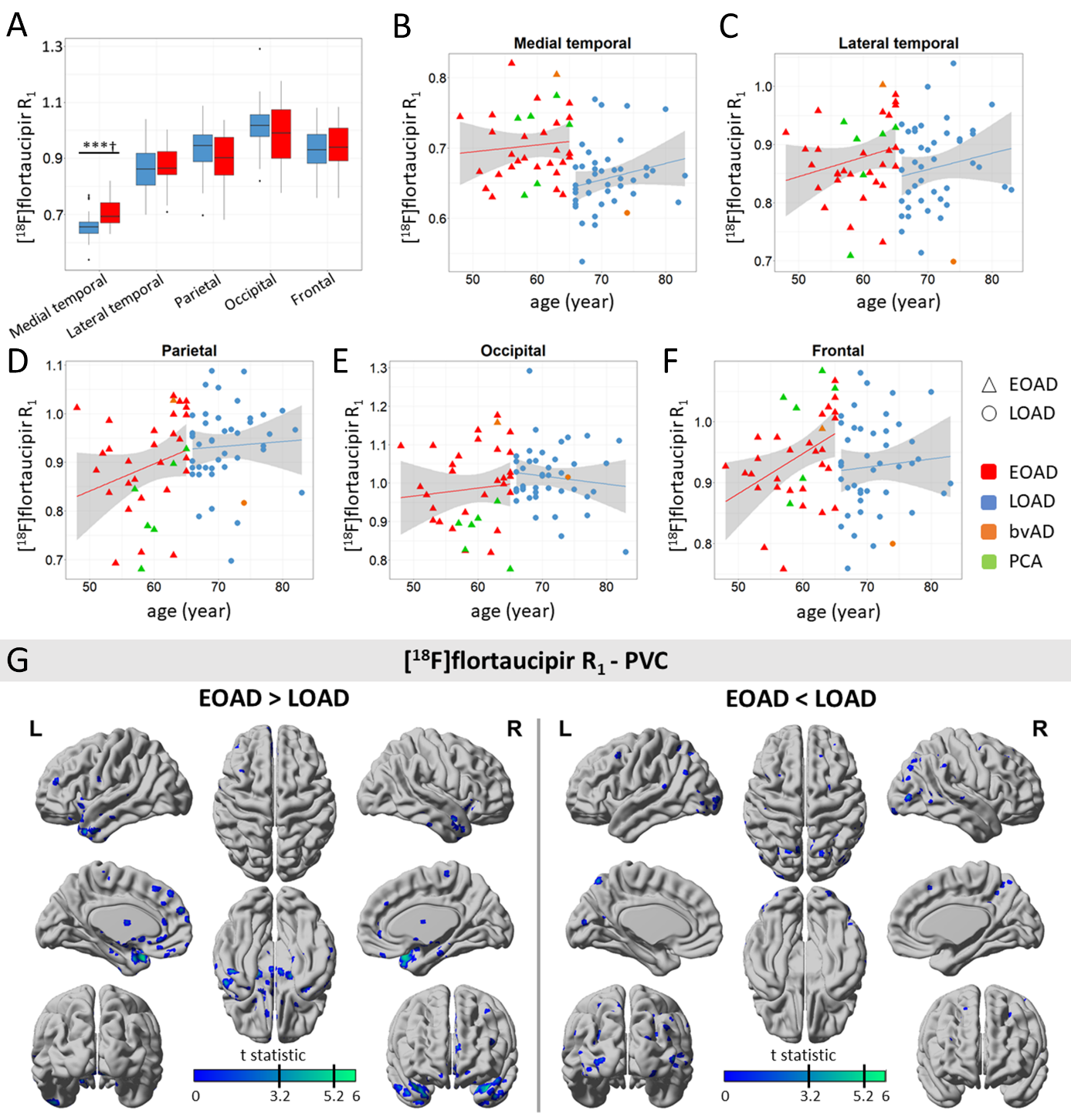


**sFigure-6. Partial volume-corrected (PVC) [^18^F]flortaucipir R_1_ for early- and late-onset AD.** A) Boxplot of partial volume-corrected [^18^F]flortaucipir R_1_ values for each region-of-interest (ROI). B-F) Scatterplots for partial volume-corrected [^18^F]flortaucipir R_1_ by age for each ROI. G) Results from voxel-wise contrast for partial volume-corrected [^18^F]flortaucipir R_1_ between early-onset AD (EOAD) and late-onset AD (LOAD). Indicated by the black lines on the color scale are thresholds for p<0.001, uncorrected (t = 3.20) and for p<0.05, FWE-corrected (t = 5.24). ***p<0.001, †p_FDR_<0.05.


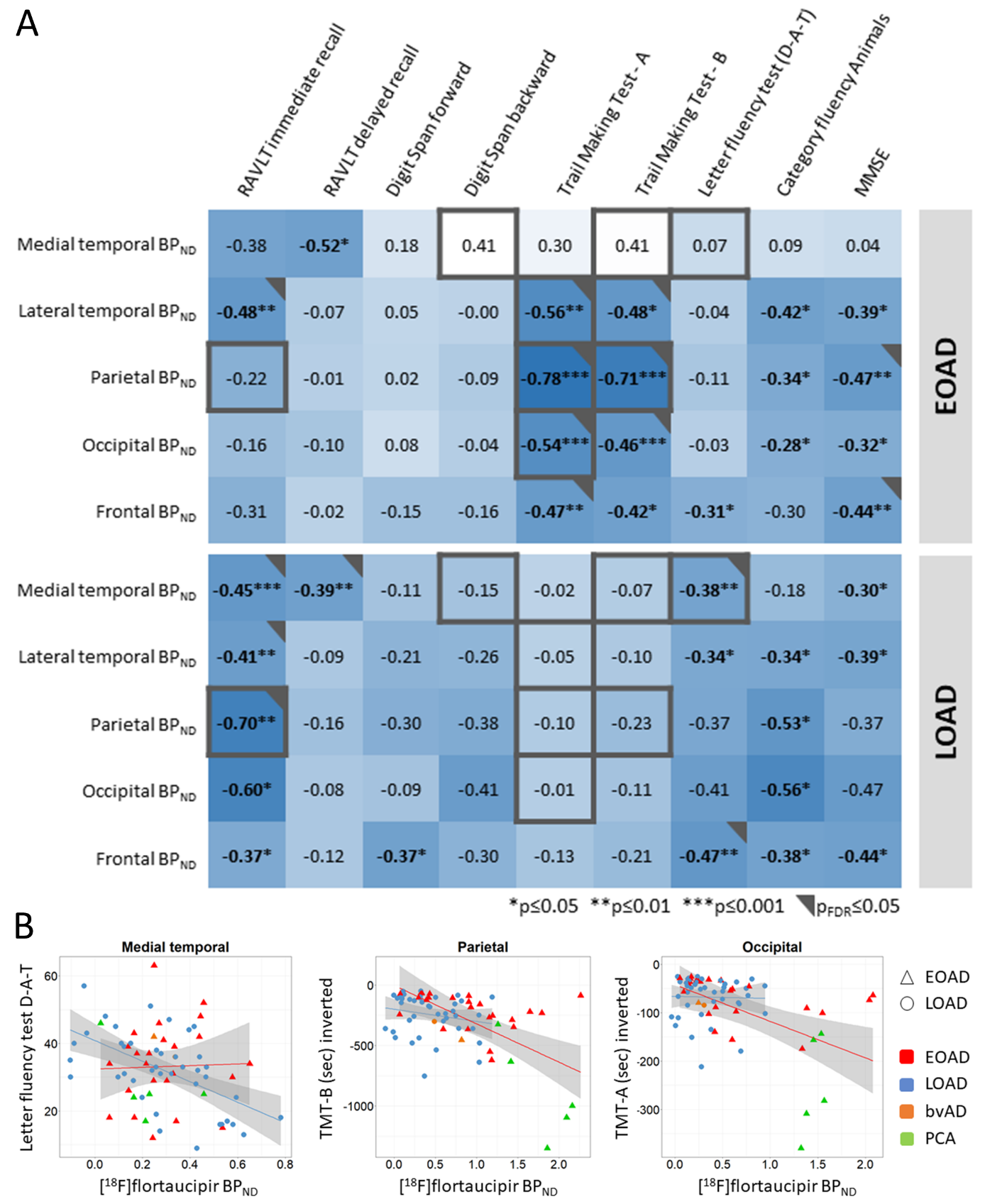


**sFigure-7. Associations between partial volume-corrected [^18^F]flortaucipir BP_ND_ and cognitive test scores for early- and late-onset AD.** A) Significant modification of age-at-onset as assessed in the model including all AD subjects from the cognition subsample (n=72), adjusted for age, sex, and education is depicted in grey (representing interaction terms at p≤0.10) and black (representing interaction terms at p_FDR_≤0.05) squares. Standardized regression coefficients are depicted for early-onset AD (EOAD) and late-onset AD (LOAD) separately. B) A selection of scatterplots for the association between partial volume-corrected [^18^F]flortaucipir BP_ND_ and neuropsychological test scores.


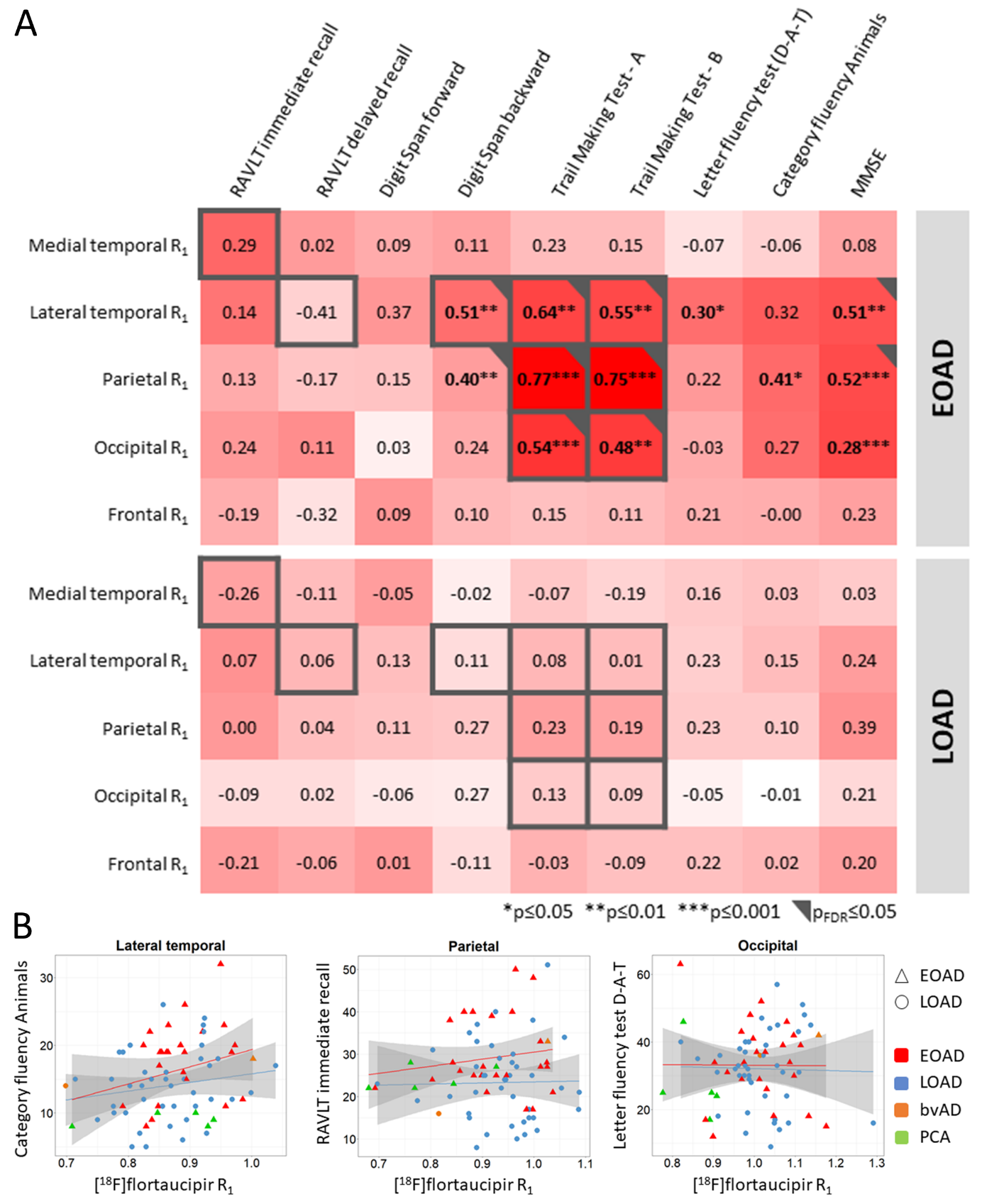


**sFigure-8. Associations between partial volume-corrected [^18^F]flortaucipir R_1_ and cognitive test scores for early- and late-onset AD.** A) Significant modification of age-at-onset as assessed in the model including all AD subjects from the cognition subsample (n=72), adjusted for age, sex, and education is depicted in grey (representing interaction terms at p≤0.10) and black (representing interaction terms at p_FDR_≤0.05) squares. Standardized regression coefficients are depicted for early- and late-onset AD separately. B) Selection of scatterplots for the association between partial volume-corrected [^18^F]flortaucipir R_1_ and neuropsychological test scores.
